# Fundal partial placenta percreta complicated with postpartum hemoperitoneum: A case report

**DOI:** 10.1016/j.ijscr.2021.106482

**Published:** 2021-10-06

**Authors:** Dema Adwan, Wessam Taifour, Rafat Bhsass, Danny Taifour

**Affiliations:** a Damascus University, Gynecology and Obstetrics Hospital, Damascus, Syria; b Faculty of Medicine, Damascus University, Damascus, Syria

**Keywords:** Fundal placenta percreta, Retained placenta, Preterm labor, Hemoperitoneum, Case report

## Abstract

**Introduction:**

The fundus of the uterus is a rare location for abnormally invasive placenta compared with the common site of abnormally invasive placenta in the lower segment of the uterus.

**Presentation of case:**

We report a case of a 38-year-old multipara woman who had a fundal partial placenta percreta with no prior cesarean sections, which presented as a retained placenta after preterm labor, and complicated with hemorrhagic shock due to postpartum hemoperitoneum, thus it was diagnosed after surgery and managed by subtotal hysterectomy.

**Discussion:**

We discuss the most common risk factors for abnormally invasive placenta and its diagnosis and management. We compare the possibility of leading to invasive placenta resulting from curettage trauma and cesarean delivery scars.

**Conclusion:**

History of uterine surgical procedures without prior cesarean delivery must raise suspicion of abnormally invasive placenta regardless of its localization, especially when associates with preterm labor or retained placenta.

## Highlights

• Placenta percreta may complicated with hemorrhagic shock due to postpartum hemoperitoneum.
• Manual removal of the retained placenta percreta may cause the rupture of its vessels leading to hemoperitoneum.
• Abnormally invasive placenta should be considered as a cause of preterm labor or retained placenta despite its localization.
• The best management of abnormally invasive placenta remains unclear.

## 1 Introduction

Abnormally invasive placenta also called placenta accrete spectrum (PAS) is a life threatening clinical condition, where the placenta attaches too deeply into the uterine wall and does not separate spontaneously, therefore sever bleeding can occur while trying to remove it. It comprises three types according to the depth of the placental invasion: accrete, increta and percreta which is the least common and the most sever type accounting only 5% of all cases. Any factor that interferes with the normal development of decidua or causes a diminution in the amount of endometrium present leads to abnormally invasive placenta [1]. A low-lying placenta on the front wall in every woman with previous cesarean delivery must raise the suspicion of abnormally invasive placenta [2]. Nevertheless, fundal localization is a rare situation of abnormally invasive placenta and is less likely to have prior cesarean delivery compared to previa placenta [3]. Regardless of its location, abnormally invasive placenta is associated with adverse maternal and neonatal outcomes, some of which are life-threatening [4].

Our work has been reported in line with the SCARE criteria [5].

## 2 Presentation of case

A 38-year-old multipara woman (gravida 4, para 2) was admitted to our hospital in November 2020 at 32 weeks of gestation experiencing preterm labor, with a blood pressure of 110/70 mm Hg, and a heart rate of 80 beats/min, her laboratory tests were normal (Hb: 11.4 g/dl, hematocrit: 31.5%, platelets count: 180 × 10^3^/μl, normal white blood cell count and TSH). She has a history of hypothyroidism treated with 100 mg levothyroxine, two miscarriages which was treated by dilatation and curettage (D&C) and a retained placenta in the last pregnancy was also treated by curettage and blood transfusion, no significant family history, she denied alcohol and tobacco use. After 10 h she had a normal vaginal delivery, but the placenta failed to deliver after 30 min and the diagnosis of retained placenta was made, manual removal was attempted unsuccessfully. An ultrasound then did not show any sign of separation, and the *clear zone* (the normal hypoechoic zone between the placenta and the myometrium) was absent. Thus, the option of leaving the placenta *in situ* was decided with consecutive observation.

**Fig. 1:**
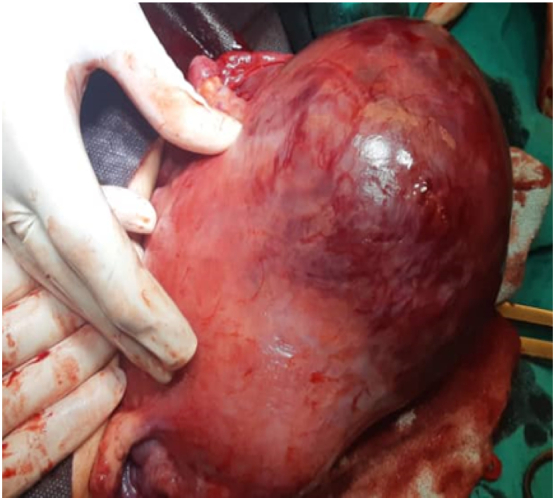
Intraoperative aspect revealing an engorged uterus during the surgery.

6 h later, her heart rate started to increase and blood pressure was 100/60 mm Hg, an emergency hemoglobin was 9.3 g/dl. Surgery was decided, Fenchtel incision was made after general anesthesia, progressive bleeding and clots were found in the abdominal cavity, the uterus was engorged with signs of placental invasion at the fundus with a rupture of one of its vessels causing the bleeding [1], [2]. A subtotal hysterectomy was performed and samples were sent for histopathology (Fig. 1).

**Fig. 2:**
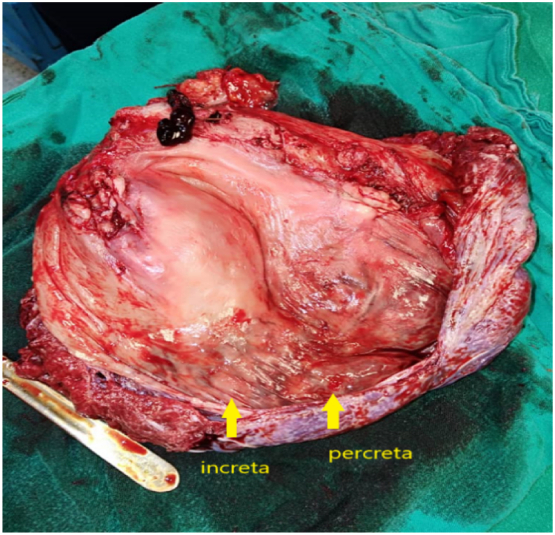
The gross pathology specimen. The yellow arrows point to the placenta where it invades the uterine wall.

**Fig. 3:**
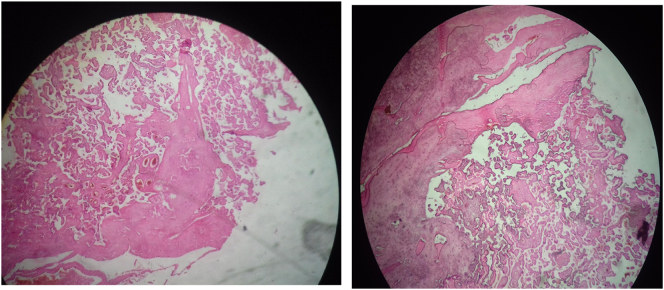
Histopathology aspect, showing chorionic villi in the myometrium of the uterus, which explains the placenta percreta.

2 full blood unites were transfused during and after the surgery. She was discharged 2 days after the surgery. The gross pathology specimen showed a site of focal placenta percreta and sites of focal placenta increta (Fig. 2). The histology of the specimen showed focal areas, of which the placenta invades the uterine serosa layer, while in other areas the placenta invades the muscular layer only (Fig. 3).

## 3 Discussion

The most common independent risk factors for abnormally invasive placenta are placenta previa and a history of previous cesarean deliveries, accordingly the risk increases with the increasing number of cesarean deliveries. Although any uterine surgical procedure (previous myomectomy, endometrial defects due to vigorous curettage, submucosal leiomyoma, thermal ablation, and uterine artery embolization) is a risk factor, the importance of these risks remains unclear [6]. Moreover, the small trauma to the uterine wall after curettage is less likely to lead to a deeper invasive placentation such as placenta percreta compared with the larger and deeper scars resulting from cesarean delivery (50% *vs* 80%) [7], [8]. There is a possibility that a fundus silent uterine defect has been caused in the last curettage and led to abnormal placenta adhesion in the current pregnancy. Most uterine perforations after curettages are not detected usually [9]. In our case, the absence of the previa and prior cesarean deliveries decreases the suspicion of the abnormally invasive placenta despite the patient has a history of 3 curettages.

The incidence of placenta accreta spectrum in the upper uterine segment consists of a small proportion of patients compared with the lower segment localization. Placenta percreta is the rarest form, representing only 5–7% of PAS. In a previous literature review in 2019, they only found 133 cases of uterine body abnormal placentation over more than 70 years, 69% of it was in the fundus and 8.3% presented with retained placenta [8]. Abnormally invasive placenta can be a cause of preterm birth, as it is unlikely that such patients progress beyond 36 weeks of gestation without bleeding [5]. In our patient, the bleeding may be attributed to the manual extraction attempt, or to the focal percreta placenta as the uterus contracts to expel the placenta, the blood vessels of the percreta part may be disrupted leading to the hemorrhage.

Diagnosing placenta accreta spectrum without previa is less likely to be done antepartum, and a high rate of severe maternal morbidity ensues [5], [8], [10], [11]. In our patient it was not observed on ultrasonography antenatally and diagnosed after delivery, albeit of the fact that ultrasound features of placenta accreta spectrum may be visible as early as the first trimester, therefor in patients with previous uterine surgical intervention, ultrasound evaluation - irrespective of the placenta location - should be done searching for abnormal sonographic features that are usually associated with abnormally invasive placenta [12].

The best management of abnormally invasive placenta remains unclear, a primary hysterectomy at the time of cesarean delivery or following failed removal of a retained placenta has been the mainstay of therapy. If there is not an excessive hemorrhage and the patient is hemodynamically stable, we can leave the placenta tissue *in situ* to preserve the uterus to maintain future fertility. Compression sutures; uterine packing; selective arterial embolization and/or balloon occlusion; and uterine and/or hypogastric artery ligation can be used to minimize blood loss. Methotrexate can be used as an adjuvant therapy though its controversial effectiveness [13], [14].

## 4 Conclusion

Abnormally invasive placenta should be considered as a cause of preterm labor or retained placenta if there is a history of uterine surgical procedures without prior cesarean delivery, regardless of its localization. Manual removal of the retained placenta percreta may cause the rupture of its vessels leading to hemorrhagic shock and hemoperitoneum, so vital signs should be monitored well.

## Consent

Written informed consent was obtained from the patient for publication of this case report and accompanying images. A copy of the written consent is available for review by the Editor-in-Chief of this journal on request.

## Provenance and peer review

Not commissioned, externally peer-reviewed.

## Ethical approval

This study is exempt from ethical approval in our institution.

## Funding

None.

## Guarantor

Dema Adwan (first author)
Wessam Taifour (corresponding author).

## Research registration number

None.

## CRediT authorship contribution statement

Dema Adwan: study concept, data collection.
Wessam Taifour: data collection, writing the paper, publishing.
Rafat Bhsass: data collection, writing the paper.
Danny Taifour: writing the paper, study design.

## Declaration of competing interest

The authors declare that they have no conflicts of interest to disclose.
